# Evaluation of carotid artery elasticity and its influencing factors in non-obese PCOS patients using a technique for quantitative vascular elasticity measurement

**DOI:** 10.3389/fendo.2024.1374718

**Published:** 2024-09-09

**Authors:** Yanli Hu, Bo Chen, Yingzheng Pan, Kewei Xing, Zhibo Xiao, Bo Sheng, Jia Li, Hongmei Dong, Furong Lv

**Affiliations:** ^1^ Department of Ultrasonography, Chongqing Health Center for Women and Children, Chongqing, China; ^2^ Department of Ultrasonography, Women and Children’s Hospital of Chongqing Medical University, Chongqing, China; ^3^ Department of Radiology, The First Affiliated Hospital of Chongqing Medical University, Chongqing, China; ^4^ Department of Ultrasonography, The First Affiliated Hospital of Chongqing Medical University, Chongqing, China; ^5^ Department of Obstetrics and Gynecology, Chongqing Health Center for Women and Children, Chongqing, China; ^6^ Department of Obstetrics and Gynecology, Women and Children’s Hospital of Chongqing Medical University, Chongqing, China; ^7^ Department of Clinical Laboratory, Chongqing Health Center for Women and Children, Chongqing, China; ^8^ Department of Clinical Laboratory, Women and Children’s Hospital of Chongqing Medical University, Chongqing, China

**Keywords:** polycystic ovary syndrome, body mass index, carotid artery elasticity, quantitative vascular elasticity, homocysteine, insulin resistance, hyperandrogenism

## Abstract

**Objectives:**

To evaluate the intima-media thickness (IMT) and elasticity of the carotid artery in non-obese polycystic ovary syndrome (PCOS) patients using a quantitative technique for vascular elasticity measurement and to explore the influencing factors.

**Methods:**

Sixty non-obese patients without metabolic and cardiovascular diseases who were diagnosed with PCOS in the Women and Children’s Hospital of Chongqing Medical University from January to December 2022 were prospectively selected (case group), and 60 healthy volunteers matched for body mass index were included as the control group. Body weight, height, heart rate, blood pressure, and waist-to-hip ratio were recorded. Fasting blood samples were drawn from the elbow vein to measure hormone levels including total testosterone (TT), sex hormone-binding globulin (SHBG), fasting plasma glucose (FPG), fasting insulin (FINS), lipids, and homocysteine (Hcy). The insulin resistance index (HOMA-IR) and free androgen index (FAI) were calculated. Ultrasound elastography was used to measure the IMT and elastic function parameters of the right carotid artery, including vessel diameter, wall displacement, stiffness coefficient, and pulse wave velocity. Differences in various parameters between the two groups were analyzed, and correlations between the carotid stiffness coefficient and other serological indicators were assessed using Spearman correlation analysis.

**Results:**

No significant differences in age, body mass index, heart rate, systolic blood pressure, and diastolic blood pressure were observed between the two groups (all P>0.05), while the waist-to-hip ratio (WHR) was higher in the case group than in the control group (P<0.05).The hormone level serological indicators TT and FAI were higher in the case group than in the control group, and SHBG was lower in the case group than in the control group (all P<0.05). The metabolism-related serum indicators LDL-C, HDL-C, FPG, triglycerides, and total cholesterol levels were not statistically different between the two groups (all P>0.05), and serum FINS, HOMA-IR, and Hcy levels were significantly higher in the case group than in the control group (all P<0.05).No significant difference in carotid artery diameter was observed between the case group and control group (P>0.05). The carotid artery displacement in the case group was significantly smaller than that in the control group (P<0.05), and carotid IMT, hardness coefficient, and pulse wave propagation velocity were greater in the case group than in the control group (all P<0.05). The carotid elastic stiffness coefficient was positively correlated with WHR, TT, SHBG, FAI, FINS, HOMA-IR and Hcy to varying extents and negatively correlated with SHBG.

**Conclusion:**

In non-obese PCOS patients with no metabolic or cardiovascular disease, the carotid stiffness coefficient was increased and correlated with indicators of hyperandrogenism, insulin resistance, and hyperhomocysteinemia.

## Introduction

Polycystic ovary syndrome (PCOS) is the most common gynecological disease in women of reproductive age, with an incidence of 5%–10%, and it leads to extremely complex endocrine and metabolic disorders, with vascular structural and functional abnormalities in the early clinical period and a significantly higher risk of complications in cardiovascular and cerebrovascular diseases in the long term (1). The prevalence of PCOS in women of reproductive age in China is 5.6%, and more than half of these patients are non-obese PCOS with a normal body mass index (BMI) (2). Because this population has a normal BMI and cardiovascular disease is not often present in the early stage, a method to assess cardiovascular and cerebrovascular disease risks early, comprehensively, and quantitatively represents an important clinical need.

The pathological basis of cardiovascular disease is atherosclerosis (AS), which can involve the arterial vascular beds of multiple organs throughout the body and cause different ischemic events (3). The carotid artery is the most commonly used ultrasound window for AS due to its superficial and fixed location and ease of detection. Two of the most important indicators assessed by ultrasound are the carotid medial intima-media thickness (CIMT) (4), which is a marker for predicting structural changes in the vascular wall, and the pulse wave propagation velocity (PWV) (5), which is important in evaluating the elasticity of large arteries.

Currently, many ultrasound techniques have been developed for evaluating the structure and elasticity of blood vessels, such as ultrafast pulse wave velocity (UFPWV), echo-tracking (ET), etc. However, the accuracy of the examination depends on the operator’s skill and is affected by blood pressure, which limits the accuracy of the examination results (6–8). The ultrasound elasticity quantitative technology used in this study is a radio frequency data processing technology based on the current approaches used in the field of artificial intelligence and deep learning medical image recognition, and the main feature is the data acquisition frame frequency. The technology offers very high accuracy and fully automated measurement, which can avoid the limitations of the traditional measurement methods affected by data processing (9, 10). In this study, we applied the technique to assess the carotid artery elasticity in non-obese PCOS patients via ultrasound quantitative parameters and analyzed correlations with plasma Hcy, glucose-lipid metabolism, and sex hormones in order to identify AS risk factors independent of obesity in PCOS patients.

## Materials and methods

### Study participants

Sixty consecutive patients aged 20–40 years with confirmed PCOS who visited the Women and Children’s Hospital of Chongqing Medical University from January to December 2022 were enrolled if they met the following inclusion criteria: normal body mass index (BMI <25 kg/m^2^) (11) and PCOS diagnosed in accordance with the 2004 Rotterdam criteria (12). During the same period, 60 healthy volunteers who had regular menstruation, normal ovarian size and structure, and normal BMI were included as the control group. Participants were excluded from either group according to the following exclusion criteria: (1) presence of any other endocrine disease; (2) acute infection and glucocorticoid use in the previous 2 weeks; (3) intake of B vitamins and folic acid within the previous 6 months; (4) presence of hypertension or liver or kidney disease; or (5) CIMT >1.0 mm on ultrasound associated with carotid plaque formation.

### Clinical data

The following data were recorded for all participants in both groups: age, heart rate, blood pressure, weight, height, waist-to-hip ratio (WHR), and BMI (weight/height^2^). The WHR is the ratio of the waist circumference (smallest circumference of waist) to the hip circumference (largest circumference of buttocks). All measurements were performed with the participant in a natural standing position with the abdomen relaxed. Before blood pressure measurement, each participant rested quietly for approximately 15–20 min in a quiet environment and did not drink alcohol, coffee, or strong tea. Blood pressure was measured in the right upper arm using a Yutu brand XJ11D desktop standard sphygmomanometer (Shanghai Medical Device Company), and the data were averaged from three repeated measurements.

For serological testing of all participants, cubital venous blood was drawn in the morning after 8–12 h of fasting and on 3rd to 5th day of the participant’s menstrual cycle (when no dominant follicles were detected by ultrasonography in amenorrhea patients). The blood samples were used to determine participants’ levels of total testosterone (TT), sex hormone binding globulin (SHBG), fasting plasma glucose (FPG), fasting insulin (FINS), homocysteine (Hcy), and lipid levels, including total cholesterol (TC), triglycerides (TG), high-density lipoprotein cholesterol (HDL-C), and low-density lipoprotein cholesterol (LDL-C). From these data, we calculated the homeostatic model assessment-insulin resistance (HOMA-IR), using the formula: HOMA-IR = FPG (mmol/L) × FINS (μU/mL)/22.5, as well as the free androgen index (FAI), using the formula: FAI = TT (nmol/L)/SHBG(nmol/L)×100%.

### Ultrasound examination

**Figure 1:**
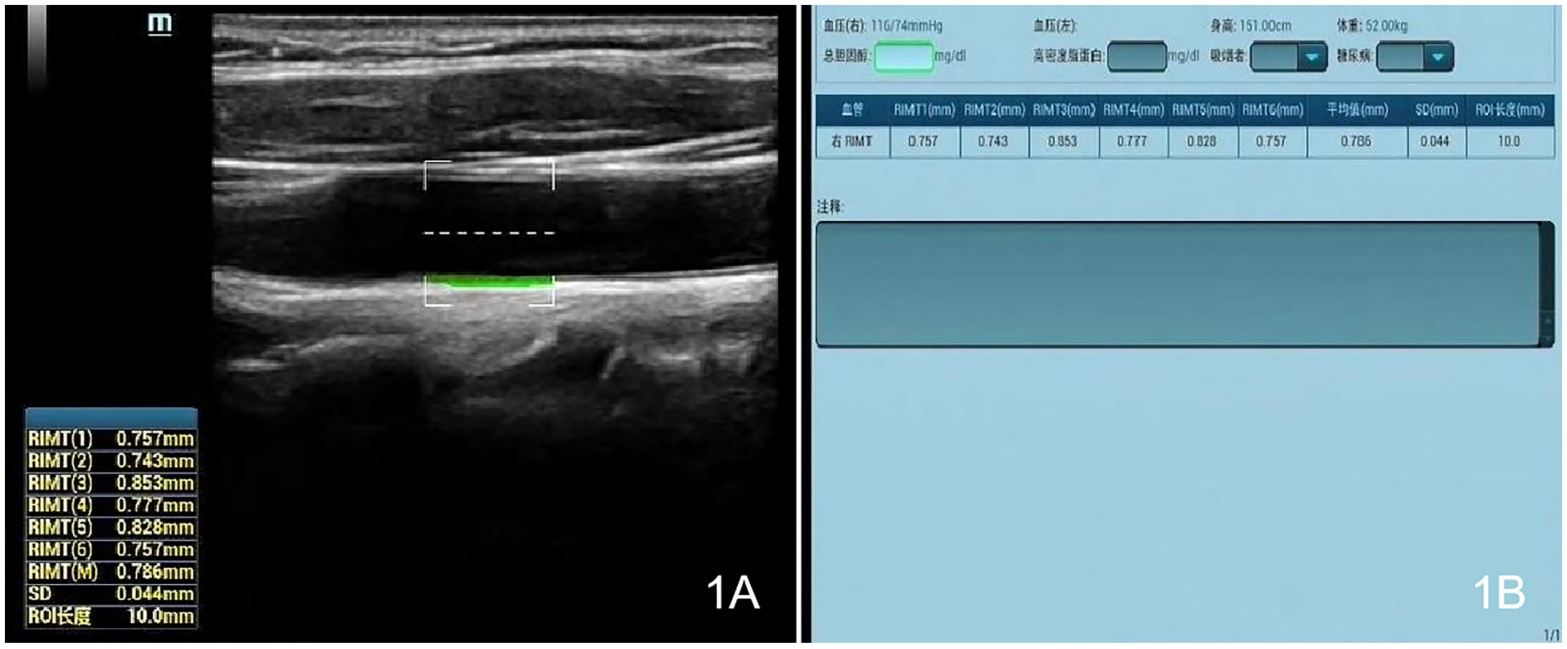
Each CIMT sampling frame was selected 1–2 cm below the bifurcation of the common carotid artery, and once the RIMT values were stable for six consecutive cardiac cycles, still images **(A)** and measurement data **(B)** were obtained.

**Figure 2:**
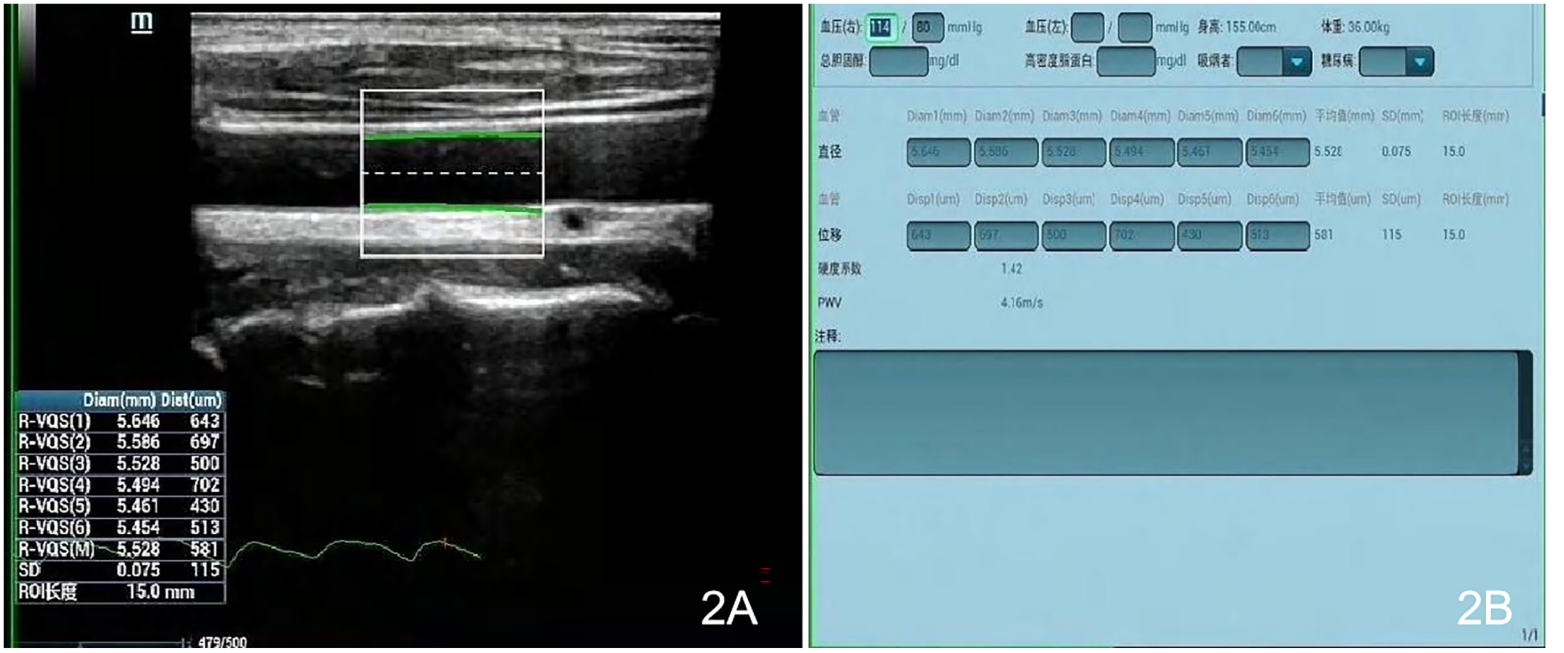
Each sampling frame was selected 1–2 cm below the bifurcation of the common carotid artery, and upon clicking the R-VQS system button, once the R-VQS analysis were stable for six consecutive cardiac cycles, the still images **(A)** and the parameters **(B)** were obtained including the carotid artery diameter (CADIA, mm), carotid artery wall displacement (CAWD, μm), stiffness coefficient (VS), and PWV (m/s).

Measurements were made with a Mindray Resona 8S color ultrasonic diagnostic apparatus (China), high-frequency linear array probe, frequency 2.5–9 MHz, and built-in digital system analysis software, including the RIMT and R-VQS systems. All participants underwent RIMT and R-VQS examination of the right carotid artery. When the acoustic beam was perpendicular to the anterior and posterior walls of the vessel and clearly showed the intima-media, the measurement points (region of interest: 15 mm) in the CIMT sampling frame were selected 1–2 cm below the bifurcation of the common carotid artery, and still images were obtained once the RIMT values were stable for six consecutive cardiac cycles. The instrument’s built-in software automatically stored and analyzed the records to produce mean radial IMT values for the common carotid artery in six cardiac cycles, along with standard deviation (SD) values (**Figures 1A, B**). Using the same operating method, R-VQS analysis results were recorded, and the parameters obtained included carotid artery diameter (CADIA, mm), carotid artery wall displacement (CAWD, μm), the stiffness coefficient (VS), and PWV (m/s), as illustrated in **Figures 2A, B**.

### Statistical analysis

Statistical analysis was performed using IBM SPSS Statistics version 21.0 (IBM Corp. Armonk, NY, USA). Data conforming to a normal distribution were expressed as mean ± SD (x ± s). When the variance was equal, t-test of two independent samples was used for comparison between the two groups. Data that did not conform to a normal distribution were expressed as median (interquartile percentages [P25, P75]). The Mann–Whitney U test was used for comparison of non-normally distributed data between the two groups. Spearman correlation analysis was performed to identify correlations between the carotid stiffness coefficient and all parameters that showed a significant difference between the PCOS and control groups. Differences and correlations for which the *P* value was <0.05 were considered statistically significant.

## Results

### Comparison of clinical data between PCOS and control groups

Among the basic clinical data recorded, only the WHR differed significantly between the PCOS and control groups, with a higher WHR observed for PCOS patients (*P*<0.05; **Table 1**). Age, BMI, heart rate, SBP and DBP did not differ significantly between the two groups (all *P*>0.05).

**Table 1:** Basic clinical characteristics of PCOS patients and control participants.

	PCOS group (n=60)	Control group (n=60)	Z	*P*
Age (years)	28 (25.00, 30.00)	28 (26.00, 32.75)	-1.640	.101
BMI (kg/m^2^)	22.31 (20.43, 23.29)	21.48 (20.76, 22.55)	-1.294	.196
WHR	0.76 (0.72, 0.81)	0.73 (0.69, 0.77)	-3.849	<.001
HR (bpm)	76.24 (67.25, 89.62)	83.36 (72.50, 89.70)	-1.265	.206
SBP (mmHg)	117.00 (110.00, 126.75)	114.96 (107.96, 125.95)	-.543	.587
DBP (mmHg)	76.00 (72.25, 86.00)	75.39 (68.96, 82.99)	-1.425	.154

BMI, body mass index; WHR, waist to hip ratio; HR, heart rate; SBP, systolic blood pressure; DBP, diastolic blood pressure.

### Comparison of serological data between PCOS and control groups

Among the hormone levels and indices assessed in this study, TT and FAI were higher in the PCOS group than in the control group, and SHBG was lower in the PCOS group than in the control group (all P<0.05; **Table 2**). No statistical differences were detected in the metabolism-related serum indicators FPG, TC, TG, LDL-C, and HDL-C levels between the two groups (all *P*>0.05), whereas the serum FINS and Hcy levels as well as the HOMA-IR were significantly higher in the PCOS group compared with the control group (all *P*<0.05; **Table 2**).

**Table 2:** Serological results for metabolism-related indicators in the PCOS and control groups.

	PCOS group (n=60)	Control group (n=60)	Z/t	P
TT(nmol/L)	2.50 (2.10, 2.88)	1.00 (0.80, 1.20)	-9.463	<.001
SHBG (nmol/L)	35.97 ± 8.59	44.26 ± 9.31	5.068	<.001
FAI	6.64 (5.36, 9.11)	2.26 (1.72, 2.93)	-9.353	<.001
FPG (mmol/L)	5.15 (4.73, 5.50)	4.90 (4.30, 5.58)	-1.587	.112
FINS (μIU/mL)	20.20 (13.05, 24.65)	6.45 (5.69, 7.59)	-7.912	<.001
Hcy (μmol/L)	11.40 (8.30, 15.98)	9.89 (7.10, 12.14)	-2.493	.013
TC (mmol/L)	4.19 (3.60, 4.78)	3.94 (3.39, 4.56)	-1.501	.133
TG (mmol/L)	2.05(1.61, 2.66)	2.03 (1.58, 2.34)	-.840	.401
LDL-C (mmol/L)	2.62 (2.32, 3.06)	2.59 (2.33, 3.06)	0.000	1.000
HDL-C (mmol/L)	1.40 (1.26, 1.57)	1.37 (1.16, 1.53)	-.916	.360
HOMA-IR	4.73 (2.76, 5.59)	1.44 (1.21, 1.69)	-7.810	<.001

TT, total testosterone; SHBG, sex hormone binding globulin; FAI, free androgen index; FPG, fasting plasma glucose; FINS, fasting insulin; Hcy, homocysteine; TC, total cholesterol; TG, triglycerides; LDL-C, low-density lipoprotein cholesterol; HDL-C, high-density lipoprotein cholesterol; HOMA-IR, homeostatic model assessment-insulin resistance.

### Carotid artery elasticity in PCOS patients and correlation with clinical characteristics

Comparison of quantitative ultrasonographic results showed no significant difference in CADIA between the PCOS group and the control group (*P*>0.05). CAWD was significantly smaller in the PCOS group than in the control group (*P*<0.05), whereas the CIMT, VS, and PWV were greater in the PCOS group than in the control group (all *P*<0.05; **Table 3**).

**Table 3:** Carotid artery elasticity parameters in PCOS patients and control participants.

	PCOS group (n=60)	Control group (n=60)	Z	*P*
RCIMT (mm)	0.60 (0.57, 0.63)	0.57 (0.55, 0.60)	-3.223	.001
CADIA (mm)	6.15 (5.78, 6.65)	6.18 (5.94, 6.67)	-1.303	.193
CAWD (μm)	396.50 (354.25, 488.25)	640.00 (522.00, 690.00)	-7.372	<.001
VS	2.68 (2.29, 3.44)	1.53 (1.39, 1.89)	-8.260	<.001
PWV (m/s)	5.66 (5.18, 6.43)	4.11 (3.39, 4.44)	-8.389	<.001

RCIMT, right carotid intima-media thickness; CADIA, carotid artery diameter; CAWD, carotid artery wall displacement; VS, stiffness coefficient; PWV, pulse wave velocity.

**Figure 3:**
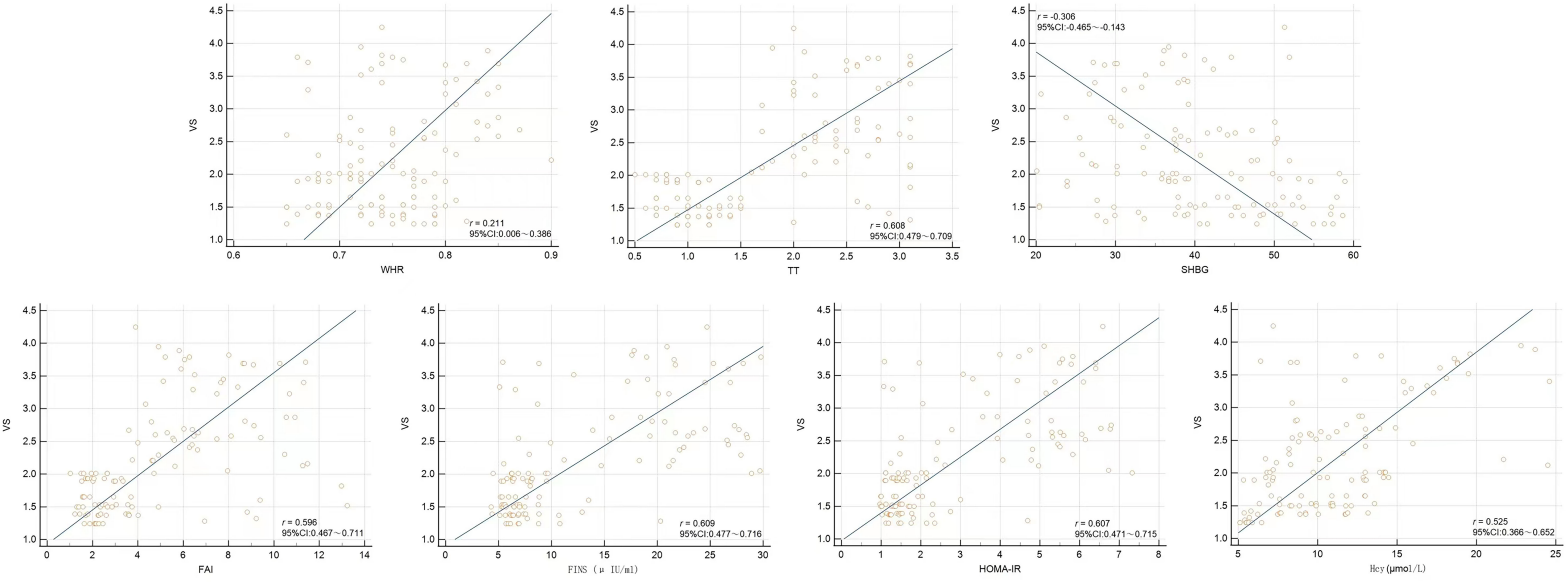
Scatter plots from Spearman correlation analysis between VS and WHR, TT, SHBG, FAI, FINS, HOMA-IR, and Hcy.

From Spearman correlation analysis, the carotid elastic stiffness coefficient was positively correlated with WHR, TT, SHBG, FAI, FINS, HOMA-IR, and Hcy to varying degrees and negatively correlated with SHBG (**Table 4** and **Figure 3**).

**Table 4:** Results of Spearman correlation analysis between VS and WHR, TT, SHBG, FAI, FINS, HOMA-IR, and Hcy.

			FINS	HOMA-IR	WHR	Hcy	TT	SHBG	FAI
*VS*	*r*		.609^**^	.607^**^	.211^*^	.525^**^	.608^**^	-.306^**^	.596^**^
	*P*		<.001	<.001	.021	<.001	<.001	.001	<.001
	95% CI	Lower Bound	.477	.471	.006	.366	.479	-.465	.467
		Upper Bound	.716	.715	.386	.652	.709	-.143	.711

**Confidence level of 0.01; the correlation is significant. *Confidence level of 0.05; there is a correlation.

FINS, fasting insulin; HOMA-IR, homeostatic model assessment-insulin resistance; WHR, waist to hip ratio; Hcy, homocysteine; TT, total testosterone; SHBG, sex hormone binding globulin; FAI, free androgen index.

## Discussion

This study investigated the changes in vascular elasticity in young, non-obese PCOS patients without traditional cardiovascular disease risk factors, and the results demonstrated that this group of patients still showed reduced vascular elasticity on carotid ultrasound elastography. Compared with an age- and weight-matched control group, the case group had significantly higher CIMT, PWV, and stiffness coefficient and significantly reduced vascular displacement (CAWD). We further explored the correlation between the stiffness coefficient and related serological indexes, and the results showed that vascular wall alterations were positively correlated with the accompanying hyperandrogenemia, insulin resistance, and hyperhomocysteinemia in PCOS patients to varying degrees. Thus, these indexes are clinically relevant for the early screening, evaluation, and follow-up of non-obese PCOS patients. This study explored the feasibility of utilizing vascular elasticity quantitative technology for non-invasive assessment of arterial vessel elasticity in non-obese patients with PCOS during the preclinical stage of atherosclerosis. The aim was to provide evidence-based clinical data to support the development of clinical preventive interventions.

Multiple studies (13–16) have demonstrated structural and functional abnormalities in the cardiovascular system in PCOS patients. The vascular changes associated with AS progress gradually, first appearing histologically as increases in collagen fibers and elastic fibers of blood vessels, with arterial wall thickening and arterial stenosis appearing later and eventually impairing the function of the perfused organs and leading to organ failure. Previous studies have suggested that the CIMT is the best ultrasound marker for detecting structural changes in the vascular wall in early AS (4). Accordingly, many studies have used the CIMT as an important indicator for evaluating the risk of AS in PCOS patients. However, Kim et al. (17) and other studies (18, 19) found that CIMT values in PCOS patients did not differ significantly from those in control groups. In a review by Alexandraki et al. (20), which retrospectively analyzed 71 studies that assessed IMT, 44 (62%) of the studies, including 2,761 patients with PCOS and 2,218 control participants, showed impairment in PCOS, whereas 27 studies, including 1,571 patients with PCOS and 1,286 control participants, did not show any difference between the groups. Such inconsistency in these findings may be related to the age of study participants, as the participants in studies that found no difference in CIMT between PCOS and control groups generally involved younger participants. Secondly, the CIMT is a marker of arterial structural change. Early changes are very small and become more significant in the later stage of AS progression. In the present study, the subjects were women of reproductive age (20–40 years old), and the CIMT values in these PCOS patients were higher than those in the control group. However, the difference was not as great as the differences in vascular elasticity parameters. In clinical studies, it is also recognized that vascular elasticity changes precede morphological changes. Thus, the detection of vascular elasticity by ultrasound is clearly more predictive in non-obese PCOS patients. The PWV in the quantitative ultrasound elasticity technique is the propagation velocity of the pressure wave in the blood vessel, and its size is directly related to the hardness of the blood vessel. This parameter was defined in the European Hypertension Guideline in 2003 (5) and holds an important position in the evaluation of the elasticity of large arteries. The CAWD can react to the degree of movement of the blood vessel, and with worsening elasticity, the displacement of the vessel is smaller. This key parameter in the quantitative ultrasound elasticity technique provides a quantitative and accurate means of measurement, that allows us to objectively assess subtle, early changes in vascular elasticity.

The findings in the present study of increased vascular stiffness are consistent with the results of most reported studies (20–23); however, a few studies still offer conflicting findings. Rees et al. (24) found that central arterial stiffness and diastolic dysfunction were not increased in young women with PCOS, whereas they were associated with both insulin resistance and central obesity. They both utilized distinct ultrasound indices to assess cardiovascular disease risk in PCOS patients, including the stiffness index (β), distensibility of the common carotid artery (CCA), and flow-mediated dilation (FMD) of the brachial artery. However, as the PCOS patients studied by these researchers had higher BMIs, arterial pressures, and basal insulinemia compared to the control participants, it remains ambiguous whether the presence of PCOS per se, rather than the comorbidities associated with this syndrome, was the primary contributor to this observed difference. This reduces the possibility that confounding factors are responsible for the differences between the populations studied, because the structure of the arterial wall slowly degrades with advancing age, hypertension, smoking, and coronary artery disease. Thus, the present study was designed to assess these markers in PCOS patients and a control population matched for age and BMI. In addition, all participants were young, normotensive, and nonsmokers with no signs or symptoms of cardiovascular disease. This study design aimed to reduce the biases stemming from population heterogeneity. Additionally, we employed advanced ultrasound imaging technology and more precise automated measurement software, thereby enhancing the accuracy and reliability of our data.

The cause of reduced arterial elasticity in PCOS is uncertain and appears to be related to the presence of individual cardiovascular risk factors. Insulin resistance plays a key role in the pathogenesis of PCOS, and while previous studies have suggested that obesity is one of the most important causes of insulin resistance, the present study found that non-obese patients with PCOS can also have insulin resistance. Insulin resistance in non-obese patients with PCOS may be related to a variety of factors such as genetics, endocrine abnormalities, inflammation, and other factors. In these patients, the cellular response to insulin becomes insensitive due to defective insulin receptors or abnormalities in the insulin signaling pathway. A chronic inflammatory state exacerbates this process and affects insulin efficacy and sensitivity. While insulin resistance is not only a metabolic abnormality in these patients, it is capable of directly leading to vascular endothelial and smooth muscle cell hypertrophy and differentiation, resulting in vascular endothelial dysfunction and vascular sclerosis (25). This is further supported by the study of Cussons et al. (26), who found that non-obese PCOS patients without concomitant insulin resistance did not have significantly altered arterial stiffness, implying an important role of insulin resistance in altered vascular stiffness.

Hyperandrogenemia is another distinguishing feature of patients with PCOS, especially in patients with a hyperandrogenemic phenotype, who have a higher prevalence of cardiovascular disease (27). Kilic et al. (28) suggested that androgen excess is independently associated with increased arterial stiffness, an association that is attributed to the ability of excess androgens to affect vascular endothelial function and to promote smooth muscle cell proliferation and migration, thereby resulting in increased vascular stiffness. This idea that hyperandrogenemia is associated with an increased risk of cardiovascular disease is supported by the present study in nonobese PCOS patients.

Recent studies have focused on the role of chronic low-grade inflammation in the pathogenesis of PCOS (29, 30). Hcy is a sulfur-containing amino acid formed during methionine metabolism that has cytotoxic effects on the vascular endothelium (31). McCully et al. (32) first proposed that Hcy plays a role in the pathogenesis of arteriosclerosis, and this was subsequently confirmed by a large number of studies in diseases such as diabetes and hypertension. However, there are limited data on the association between high Hcy and AS in women with PCOS. One study (33) showed that high Hcy (H-Hcy) levels are positively and independently associated with elevated brachial and ankle pulse wave velocity (baPWV) in female patients with PCOS, suggesting that Hcy may play a role in the pathologic process of AS in women with PCOS. However, further studies in non-obese patients with PCOS were not conducted. We found that non-obese PCOS patients have a relatively higher Hcy that is positively correlated with the VS. Long-term high Hcy status increases oxidative stress and weakens the antioxidant response, which directly or indirectly damages vascular endothelial cells, promotes smooth muscle cell proliferation, changes blood coagulation status, and impairs platelet function, thus causing vascular damage and increasing the risk of long-term cardiovascular disease in PCOS patients (34, 35).

Obesity is also recognized as an independent risk factor for As. In the present study, the WHR was higher in non-obese PCOS patients than in control participants, indicating that even for patients with a normal BMI, abdominal obesity may still exist. Dumesic et al (36) suggested that intra-abdominal fat deposition in non-obese PCOS patients may be related to hyperandrogenism. Normal weight PCOS patients exhibit preferential intra-abdominal fat storage and have an increased number of small subcutaneous abdominal adipocytes, which could constrain subcutaneous adipose storage and promote metabolic dysfunction. For non-obese PCOS patients, the WHR can better reflect the obesity status and body fat distribution of non-obese PCOS patients compared with BMI, which may explain why it was found to be a more useful indicator for predicting the risk of AS. While healthy weight management may be important in treating PCOS patients, improving abdominal obesity should also be a goal in the health management of these patients.

The present study has some limitations. This study was a single-center, small-sample, cross-sectional study with possible bias, and there was no further categorization of different clinical phenotypes of patients with PCOS. Consequently, there remains a need for long-term, large-scale, prospective studies in PCOS patients, particularly focusing on cardiovascular outcomes across different PCOS phenotypes, to better understand the impact of PCOS on cardiovascular function. Future research could build upon the present findings by incorporating more datasets and expanding the sample size.

In summary, female patients with PCOS and normal BMI showed alterations in vessel wall thickness and elasticity even in the absence of traditional AS risk factors, indicating early signs of AS. These changes are associated with hyperandrogenism, insulin resistance, hyperhomocysteinemia, and abdominal obesity. Clinical attention should be paid to the risk factors for early-onset AS in non-obese PCOS patients and included in patient health management programs to slow the atherosclerotic process.

## Data Availability

The raw data supporting the conclusions of this article will be made available by the authors, without undue reservation.

## References

[1] Marciniak A Nawrocka Rutkowska J Brodowska A Wiśniewska B Starczewski B. Cardiovascular system diseases in patients with polycystic ovary syndrome - the role of inflammation process in this pathology and possibility of early diagnosis and prevention. Ann Agric Environ Med. (2016) 23(4):537–41. doi: 10.5604/12321966.1226842 28030919

[2] Li R Zhang Q Yang D Li S Lu S Wu X. Prevalence of polycystic ovary syndrome in women in China: a large community-based study. Hum Reprod. (2013) 28(9):2562–9. doi: 10.1093/humrep/det262 23814096

[3] Talari HR Azad ZJ Hamidian Y Samimi M Gilasi HR Ebrahimi Afshar F. Effects of carnitine administration on carotid intima-media thickness and inflammatory factors in patients with polycystic ovary syndrome: a randomized, double-blind, placebo-controlled trial. Int J Prev Med. (2019) 7:10–89. doi: 10.4103/ijpvm.IJPVM_2_18 PMC6592103 31360336

[4] Liu F Ma H Ma Y Zhou W Wang C Xiong Y. The correlation between serum sclerostin level and arterial stiffness in peritoneal dialysis patients. Evid Based Complement Alternat Med. (2022) 2022:4247782. doi: 10.1155/2022/4247782 35990820 PMC9385280

[5] European Society of Hypertension-European Society of Cardiology Guidelines Committee. 2003 European Society of Hypertension-European Society of Cardiology guidelines for the management of arterial hypertension. J Hypertens. (2003) 21:1011–53. doi: 10.1097/00004872-200306000-00001 12777938

[6] Alis D Durmaz ESM Civcik C Tutuncu M Saip S Kocer N. Assessment of the common carotid artery wall stiffness by Shear Wave Elastography in Behcet's disease. Med Ultrason. (2018) 20:446–52. doi: 10.11152/mu-1565 30534651

[7] Hae Kim C Wang S Park JB Jung KH E Yoon Y Lee SP. Assessing impact of high-dose pitavastatin on carotid artery elasticity with speckle-tracking strain imaging. J Atheroscler Thromb. (2018) 25:1137–48. doi: 10.5551/jat.42861 PMC6224202 29515050

[8] Ma X Zhu Z Wang Y Shen B Jiang X Liu W. Quantifying carotid stiffness in a pre-hypertensive population with ultrafast ultrasound imaging. Ultrasonography. (2023) 42:89–99. doi: 10.14366/usg.22039 36588181 PMC9816694

[9] Chen YA Chen PY Lin SK. Three-dimensional ultrasound for carotid vessel wall volume measurement. Tzu Chi Med J. (2021) 34:88–94. doi: 10.4103/tcmj.tcmj_283_20 35233362 PMC8830545

[10] Malik AEF Giudici A van der Laan KWF Op 't Roodt J Mess WH Delhaas T. Detectable bias between vascular ultrasound echo-tracking systems: relevance depends on application. J Clin Med. (2022) 12:69. doi: 10.3390/jcm12010069 36614870 PMC9821692

[11] Guilbert JJ. The world health report 2002 - reducing risks, promoting healthy life. Educ Health. (2003) 16:230. doi: 10.1080/1357628031000116808 14741909

[12] Rotterdam ESHRE/ASRM-Sponsored PCOS Consensus Workshop Group. Revised 2003 consensus on diagnostic criteria and long-term health risks related to polycystic ovary syndrome. Fertil Steril. (2004) 81:19–25. doi: 10.1016/j.fertnstert.2003.10.004 14711538

[13] Christian RC Dumesic DA Behrenbeck T Oberg AL Sheedy PF 2nd Fitzpatrick LA. Prevalence and predictors of coronary artery calcification in women with polycystic ovary syndrome. J Clin Endocrinol Metab. (2003) 88:2562–8. doi: 10.1210/jc.2003-030334 12788855

[14] Orio F Jr Palomba S Cascella T De Simone B Di Biase S Russo T. Early impairment of endothelial structure and function in young normal-weight women with polycystic ovary syndrome. J Clin Endocrinol Metab. (2004) 89:4588–93. doi: 10.1210/jc.2003-031867 15356067

[15] Cussons AJ Stuckey BG Watts GF. Cardiovascular disease in the polycystic ovary syndrome: new insights and perspectives. Atherosclerosis. (2006) 185:227–39. doi: 10.1016/j.atherosclerosis.2005.10.007 16313910

[16] Yalcin Bahat P Özel A Demirci A. Evaluation of carotid artery intima-media thickness as a cardiovascular risk factor in patients with polycystic ovary syndrome. Cureus. (2021) 13:e13025. doi: 10.7759/cureus.13025 33542889 PMC7849912

[17] Kim JJ Choi YM Kang JH Hwang KR Chae SJ Kim SM. Carotid intima-media thickness in mainly non-obese women with polycystic ovary syndrome and age-matched controls. Obstet Gynecol Sci. (2013) 56:249–55. doi: 10.5468/ogs.2013.56.4.249 PMC3784140 24328010

[18] Barcellos CR Lage SH Rocha MP Hayashida SA Baracat EC Romano A. Polycystic ovary syndrome and obesity do not affect vascular parameters related to early atherosclerosis in young women without glucose metabolism disturbances, arterial hypertension and severe abnormalities of lipid profile. Gynecol Endocrinol. (2013) 29:370–4. doi: 10.3109/09513590.2012.743009 23327607

[19] Buyukkaya R Besir FH Yazgan S Karatas A Kose SA Aydin Y. The evaluation of carotid intima-media thickness and visceral obesity as an atherosclerosis predictor in newly-diagnosed polycystic ovary syndrome. Clin Ter. (2014) 165:e6–11. 24589963

[20] Alexandraki KI Kandaraki EA Poulia KA Piperi C Papadimitriou E Papaioannou TG. Assessment of early markers of cardiovascular risk in polycystic ovary syndrome. touchREV Endocrinol. (2021) 17(1):37–53. doi: 10.17925/EE.2021.17.1.37 35118445 PMC8320007

[21] Soares GM Vieira CS Martins WP Franceschini SA dos Reis RM Silva de Sá MF. Increased arterial stiffness in nonobese women with polycystic ovary syndrome (PCOS) without comorbidities: one more characteristic inherent to the syndrome? Clin Endocrinol (Oxf). (2009) 71(3):406–11. doi: 10.1111/j.1365-2265.2008.03506.x 19094071

[22] Talbott EO Zborowski JV Rager JR Boudreaux MY Edmundowicz DA Guzick DS. Evidence for an association between metabolic cardiovascular syndrome and coronary and aortic calcification among women with polycystic ovary syndrome. J Clin Endocrinol Metab. (2004) 89(11):5454–61. doi: 10.1210/jc.2003-032237 15531497

[23] Brinkworth GD Noakes M Moran LJ Norman R Clifton PM. Flow-mediated dilatation in overweight and obese women with polycystic ovary syndrome. BJOG. (2006) 113(11):1308–14. doi: 10.1111/j.1471-0528.2006.01090.x 17059392

[24] Rees E Coulson R Dunstan F Evans WD Blundell HL Luzio SD. Central arterial stiffness and diastolic dysfunction are associated with insulin resistance and abdominal obesity in young women but polycystic ovary syndrome does not confer additional risk. Hum Reprod. (2014) 29(9):2041–9. doi: 10.1093/humrep/deu180 25035436

[25] Arcaro G Cretti A Balzano S Lechi A Muggeo M Bonora E. Insulin causes endothelial dysfunction in humans: sites and mechanisms. Circulation. (2002) 105:576–82. doi: 10.1161/hc0502.103333 11827922

[26] Cussons AJ Watts GF Stuckey BG. Dissociation of endothelial function and arterial stiffness in nonobese women with polycystic ovary syndrome (PCOS). Clin Endocrinol (Oxf). (2009) 71:808–14. doi: 10.1111/j.1365-2265.2009.03598.x 19508597

[27] Carmina E Chu MC Longo RA Rini GB Lobo RA. Phenotypic variation in hyperandrogenic women influences the findings of abnormal metabolic and cardiovascular risk parameters. J Clin Endocrinol Metab. (2005) 90:2545–9. doi: 10.1210/jc.2004-2279 15728203

[28] Kilic D Kilic ID Sevgican CI Kilic O Alatas E Arslan M. Arterial stiffness measured by cardio-ankle vascular index is greater in non-obese young women with polycystic ovarian syndrome. J Obstet Gynecol Res. (2021) 47:521–8. doi: 10.1111/jog.14543 33145911

[29] Rudnicka E Suchta K Grymowicz M Calik-Ksepka A Smolarczyk K Duszewska AM. Chronic low grade inflammation in pathogenesis of PCOS. Int J Mol Sci. (2021) 22:3789. doi: 10.3390/ijms22073789 33917519 PMC8038770

[30] Caglar GS Oztas E Karadag D Pabuccu R Demirtas S. Ischemia-modified albumin and cardiovascular risk markers in polycystic ovary syndrome with or without insulin resistance. Fertil Steril. (2011) 95:310–3. doi: 10.1016/j.fertnstert.2010.06.092 20701906

[31] Selhub J Miller JW. The pathogenesis of homocysteinemia: interruption of the coordinate regulation by S-adenosylmethionine of the remethylation and transsulfuration of homocysteine. Am J Clin Nutr. (1992) 55:131–8. doi: 10.1093/ajcn/55.1.131 1728812

[32] McCully KS. Vascular pathology of homocysteinemia: implications for the pathogenesis of arteriosclerosis. Am J Pathol. (1969) 56:111–28. PMC2013581 5792556

[33] Wu X Li Z Sun W Zheng H. Homocysteine is an indicator of arterial stiffness in Chinese women with polycystic ovary syndrome. Endocr Connect. (2021) 10(9):1073–9. doi: 10.1530/EC-21-0224 PMC8428028 34355700

[34] van Bergen En Henegouwen K Hutten BA Luirink IK Wiegman A de Groot E Kusters DM. Intima-media thickness in treated and untreated patients with and without familial hypercholesterolemia: A systematic review and meta-analysis. J Clin Lipidol. (2022) 16:128–42. doi: 10.1016/j.jacl.2022.01.009 35184975

[35] Cerqueira JM Costa LO Nogueira Ade A Silva DC Torres Dde O Santos AC. Homocisteinemia em mulheres com síndrome dos ovários policísticos [Homocysteinemia in polycystic ovary syndrome women. Rev Bras Ginecol Obstet. (2010) 32:126–32. doi: 10.1590/S0100-72032010000300005 20512259

[36] Dumesic DA Akopians AL Madrigal VK Ramirez E Margolis DJ Sarma MK. Hyperandrogenism accompanies increased intra-abdominal fat storage in normal weight polycystic ovary syndrome women. J Clin Endocrinol Metab. (2016) 101:4178–88. doi: 10.1210/jc.2016-2586 PMC5095243 27571186

