# Corrigendum: Evaluation of carotid artery elasticity and its influencing factors in non-obese PCOS patients using a technique for quantitative vascular elasticity measurement

**DOI:** 10.3389/fendo.2024.1529178

**Published:** 2024-12-10

**Authors:** Yanli Hu, Bo Chen, Yingzheng Pan, Kewei Xing, Zhibo Xiao, Bo Sheng, Jia Li, Hongmei Dong, Furong Lv

**Affiliations:** ^1^ Department of Ultrasonography, Chongqing Health Center for Women and Children, Chongqing, China; ^2^ Department of Ultrasonography, Women and Children’s Hospital of Chongqing Medical University, Chongqing, China; ^3^ Department of Radiology, The First Affiliated Hospital of Chongqing Medical University, Chongqing, China; ^4^ Department of Ultrasonography, The First Affiliated Hospital of Chongqing Medical University, Chongqing, China; ^5^ Department of Obstetrics and Gynecology, Chongqing Health Center for Women and Children, Chongqing, China; ^6^ Department of Obstetrics and Gynecology, Women and Children’s Hospital of Chongqing Medical University, Chongqing, China; ^7^ Department of Clinical Laboratory, Chongqing Health Center for Women and Children, Chongqing, China; ^8^ Department of Clinical Laboratory, Women and Children’s Hospital of Chongqing Medical University, Chongqing, China

**Keywords:** polycystic ovary syndrome, body mass index, carotid artery elasticity, quantitative vascular elasticity, homocysteine, insulin resistance, hyperandrogenism

In the published article, there was an error in the order of the affiliations of Hongmei Dong. Instead of “Hongmei Dong^1,2*^”, it should be “Hongmei Dong^2,1*^”.

The authors apologize for this error and state that this does not change the scientific conclusions of the article in any way. The original article has been updated.

